# Sustained protective rabies neutralizing antibody titers after administration of cationic lipid-formulated pDNA vaccine

**DOI:** 10.1186/1479-0556-4-2

**Published:** 2006-02-15

**Authors:** Michal Margalith, Adrián Vilalta

**Affiliations:** 1 Vical Incorporated, 10390 Pacific Center Ct, San Diego, CA 92121, USA

## Abstract

Published data indicate that formulation of pDNA with cationic lipids could greatly enhance the response to a pDNA vaccine in larger mammals. The present work tested the influence of several pDNA:cationic lipid formulations on rabies neutralizing titers. Plasmid expressing Rabies G protein (CVS strain) was evaluated in vivo for ability to elicit neutralizing titers. pDNA:DMRIE-DOPE formulated at two DNA:cationic lipid molar ratios was compared in mice to a Vaxfectin™-pDNA formulation. Mouse data indicate that Vaxfectin™ is more effective than DMRIE-DOPE in eliciting neutralizing titers. In addition, the ratio of pDNA to DMRIE-DOPE can also affect neutralizing titers. Our data show that sustained neutralizing titers (120 days) can be obtained after a single administration of DMRIE-DOPE-formulated pDNA in rabbits.

## Introduction

Each year rabies kills more than 50,000 people and millions of animals worldwide [1]. Although vaccination of dogs, elimination of strays and control of wild animals have been effective in preventing most cases of human rabies in North America and Western Europe, the same approaches have been difficult to implement in parts of the developing world because of cultural, economic and political reasons. Therefore, a pressing need for safe, effective and affordable human rabies vaccines still exists in some developing countries. Plasmid DNA (pDNA) vaccines could fill this gap due to their demonstrated safety, speed and ease of development, and their inherent stability allowing for long-term stockpiling and simplified distribution.

Rabies vaccines of nerve tissue origin have been available for post-exposure use in humans since the end of the 19^th ^century. These vaccines are inexpensive but of relatively low potency requiring a difficult vaccination schedule of 14 to 21 inoculations with two booster doses for post-exposure treatments. In addition, serious side effects are frequently associated with these vaccines, which limit their suitability for pre-exposure prophylaxis. Many developing countries still depend on nerve tissue-derived vaccines. Safe and highly efficacious rabies vaccines produced in cell culture have been available for almost 30 years in affluent countries. Three rabies vaccines are currently available in the United States for human use: human diploid cell vaccine (HDCV); rabies vaccine adsorbed (RVA); and purified chick embryo cell culture vaccine (PCECV). All three vaccines can be used in both pre- and post-exposure applications. In spite of the efficacy and safety profile of cell culture vaccines, manufacturing, storage and distribution costs make them prohibitive for widespread use in many poorer countries. Other rabies vaccine approaches under evaluation include the use of vaccinia virus [2-4] and pDNA [5,6] to deliver rabies antigens.

The feasibility of pDNA rabies vaccines has been demonstrated in several animal models including mice [5], dogs [7], cats [8], horses [9] and non-human primates [6,10]. Interestingly, a single administration of rabies pDNA vaccine in mice was as effective as five doses of a cell culture derived vaccine [11]. Several routes of administration have been tested including intramuscular [5,9], intradermoplantar [12] and intradermal [5,7]. pDNA has also been delivered using the Biolistic^® ^system [13] as well as by needle injection. Rabies pDNA vaccines have been delivered in PBS [9,11], Adju-Phos^® ^(aluminum phosphate) [9], monophosphoryl lipid A [5] as well as cationic lipid formulations [9]. The choice of formulation can be critical for the effectiveness of the vaccine, as shown by Fischer and co-workers [9] who demonstrated that a cationic lipid-formulated rabies pDNA vaccine was more effective in eliciting a humoral response than aluminum phosphate or PBS formulation. The proof of concept of pDNA rabies vaccines in animal models suggests that development of an effective pDNA vaccine for human immunization should be achievable.

Protection against rabies infection depends primarily on humoral immunity [14]. Intramuscular injection of pDNA in PBS has been shown to elicit potent cellular but relatively low antibody responses [15,16]. Therefore, the use of formulations that drive robust antibody responses would be expected to improve the performance of a pDNA rabies vaccine beyond that achievable with pDNA in PBS. Complexation of immunogen-encoding pDNA with cationic lipid systems, such as DMRIE-DOPE and Vaxfectin™ [15] offers a potential enhancement to the relatively weak humoral response elicited by pDNA in PBS. For instance, Hartikka and co-workers [17] showed a 20-fold increase in antibody titers against influenza nucleoprotein when formulating pDNA with Vaxfectin™ compared to titers obtained with pDNA in PBS. Recently, Hermanson and co-workers [18] demonstrated the successful protection of rabbits against a lethal dose of aerosolized anthrax spores after immunization with a pDNA:DMRIE-DOPE vaccine. Fischer and collaborators [9] were able to elicit protective titers in horses after a single injection of pDNA:DMRIE-DOPE. In addition, Fischer demonstrated that titers obtained after administration of rabies pDNA:cationic lipid were higher than those obtained after administration of pDNA:Adju-Phos^® ^and no detectable antibodies were obtained after one injection of pDNA in PBS.

Previous published work [9,19] indicates that pDNA:cationic lipid formulations could prove successful in raising protective rabies antibody titers in larger mammals. Particularly, Fischer's data on horse pDNA vaccinations [9] indicate that DMRIE-DOPE could be used as a component of such a vaccine; moreover, pDNA:DMRIE-DOPE vaccines are currently being evaluated in human trials [20]. However, there are no published data on the effect of different cationic lipid formulations on rabies titers. Our results suggest that the cationic lipid Vaxfectin™ appears to be a better candidate than DMRIE-DOPE for formulating a pDNA rabies vaccine. In addition, higher titers were observed at low vaccine doses in mice when DMRIE-DOPE was used at a 4:1 molar ratio (pDNA:cationic lipid) as opposed to the 2.5:1 ratio reported previously [9]. Vaccination of rabbits with pDNA:DMRIE-DOPE resulted in high levels of neutralizing antibodies that persisted for a minimum of 195 days; moreover, data from rabbit studies indicate that protective titers can be obtained with low doses (10 μg) of pDNA:cationic lipid. The fact that protective antibodies were observed 21 days after pDNA administration for all the cationic lipid formulations tested supports the use of rabies pDNA vaccine for post-exposure therapy. In summary, our data suggest that the choice of cationic lipid system as well as the pDNA:cationic lipid ratios can offer significant enhancement to the humoral response to rabies pDNA vaccines.

## Materials and methods

### pDNA and formulations

DNA coding for wild type rabies glycoprotein G (G protein; CVS strain) was subcloned from plasmid pKB3-JE13 [22] (Clone #40280; ATCC, Manassas, VA) into expression plasmid VR1051 using the Eco RI restriction site. The resulting plasmid was designated VR7203. VR1051 is similar to the VR1055 construct previously described [22], except for the multiple cloning site. G protein expression was tested *in vitro *by transfecting VM92 (murine melanoma cells; Vical Inc., San Diego, CA) cells with VR7203 using lipofection. The presence of G protein in transfection supernatants was confirmed by Western blot analysis using an anti-rabies monoclonal antibody (QED Bioscience, cat# 20501; San Diego, CA)(data not shown).

DMRIE-DOPE consists of a 1:1 molar ratio of DMRIE: (±)-N-(2-Hydroxyethyl)-N,N-dimethyl-2,3-bis(tetradecyloxy)-1-propanaminium bromide and DOPE: 1,2-Dioleoyl-sn-glycero-3-phosphoethanolamine [23]. The DMRIE-DOPE lipid mixture was diluted to 1.5 mM with sterile water and added to an equal volume of pDNA (2 mg/mL in 2× PBS) and vortexed briefly. The final DNA: cationic lipid molar ratio of formulation was either 4:1 or 2.5:1, depending on the test group.

Vaxfectin™ consists of an equimolar mixture of VC1052 ((±)-N- (3-aminopropyl)-N,N-dimethyl-2, 3-bis (myristoleyloxy)-1-propanaminium bromide) and DPyPE (Diphytanoylphosphatidyl-ethanolamine). Preparation of Vaxfectin™ and its formulations is described in detail by Hartikka and co-workers [17]. Briefly, the molar ratio of pDNA:cationic lipid of Vaxfectin™ formulation is 4:1. pDNA is dissolved in 0.9% NaCl, 20 mM Na phosphate (pH 7.2) at double the final concentration. Vaxfectin™ is suspended from a dry film state in 0.9% NaCl at double the final concentration.

### Animal procedures

All animal procedures were approved by Vical's IACUC and complied with the standards set forth in the *Guide for the Care and Use of Laboratory Animals *(U.S. Department of Health Services, NIH; Bethesda, MD) and the USDA's (Washington, DC) *Animal Welfare Act and Animal Welfare Regulations*.

Groups of ten, six- to eight-week old female BALB/c mice (Jackson Labs; Bar Harbor ME) were vaccinated by bilateral intramuscular injection of cationic lipid-formulated pDNA (either 2 or 5 μg; 50 μL per leg) into the quadriceps muscle group. Mice were bled at days 1 and 21 by orbital sinus puncture. The final bleed was done by cardiac puncture 48 days after pDNA administration. Serum samples were tested for neutralizing antibodies by the Rapid Fluorescence Focus Inhibition Test (RFFIT).

Groups of three New Zealand white rabbits (Harlan Sprague-Dawley; Oxford, MI), received plasmid VR7203 formulated with DMRIE-DOPE (4:1 DNA:cationic lipid molar ratio) in PBS in a total volume of 0.5 mL per intramuscular injection (*rectus femoris *bilateral injection); pDNA doses ranged from 10 μg to 1 mg, depending on the experiment. Positive control groups received a single dose of IMRAB^® ^3 (Merial, Lyon, France), a commercially available rabies veterinary vaccine. Animals were bled at regular intervals until the end of the study. Serum was tested for rabies neutralizing titers by RFFIT.

### Neutralizing titer determination and interpretation

Rabies neutralizing titers were determined by Rapid Fluorescence Focus Inhibition Test (RFFIT) at Atlanta Health Associates (Cumming, GA) using rabies CVS and standard assay techniques. Briefly, dilutions of heat-inactivated serum are incubated with a fixed amount of rabies virus (CVS strain) for 60–90 min at 37°C. Residual virus infectivity is then determined by inoculating cell cultures with the virus. Foci of virus infected cells are stained and detected by fluorescence and counted. According to the World Health Organization's Expert Rabies Committee [1] and the United States Centers for Disease Control and Prevention an antibody level of > 0.5 International Units (IU)/mL of serum indicates a positive protective antibody response to vaccination.

### Statistical analysis

Statistical analyses were conducted with SAS computer software (SAS Institute, Cary, NC). A general linear model (GLM) was performed with formulation, dose as the class variables and experimental data as the dependent variable. The interaction term of formulation dose was examined in the GLM model. Tukey method and contrast statement were used for the group-wise comparisons. Statistical significance was determined at α = 0.05 level with 2-sided test.

## Results

### Vaccination of rabbits with rabies G protein pDNA results in sustained high protective neutralizing titers

**Figure 1:**
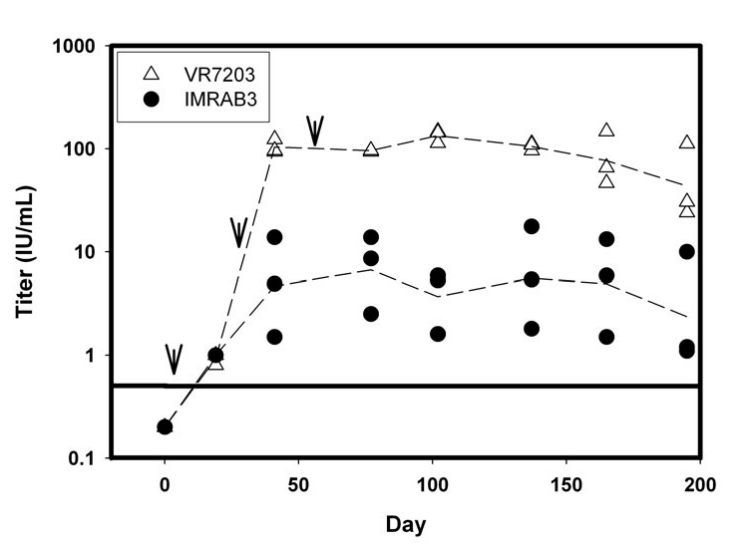
**Sustained High Protective Titers after pDNA vaccination**. Two groups of three rabbits were vaccinated with either 1 mg of rabies G protein pDNA VR7203 (Days 1, 28 and 56) (Δ) or with a single administration of IMRAB^® ^3 (●) as described in Materials and Methods. pDNA:cationic lipid molar ratio was kept at 4:1. Data points represent individual animal titers. Lines connect mean titer values for each group at each time point. Protective titer threshold (0.5 IU/mL) is indicated with a horizontal line. Vaccination days are indicated with downward arrows.

Groups of three rabbits were vaccinated with either the DMRIE-DOPE-formulated VR7203 plasmid (4:1 DNA:cationic lipid ratio) or the conventional rabies vaccine, IMRAB^® ^3. Rabbits in the pDNA group were vaccinated on Days 1, 28 and 56 with 1 mg (bilateral injection; 500 μg/500 μL per leg) of DMRIE-DOPE-formulated pDNA and were bled at regular intervals for serum collection. Animals in the IMRAB^® ^3 group received a single administration of the vaccine (1 mL) and were bled on the same schedule as the pDNA group. Serum from all animals had titers (Figure 1) higher than the 0.5 IU/mL threshold by day 21. The serum of rabbits vaccinated with pDNA reached mean titers of about 100 IU/mL by day 41. Titers in the pDNA group remained high for the duration of the study (195 Days) dropping to about 50 IU/mL at the conclusion of the study. Animals in the control group presented titers in the 4 to 8 IU/mL range after the Day 21 bleed.

### Single administration of rabies G protein pDNA results in high protective titers in rabbits

**Figure 2:**
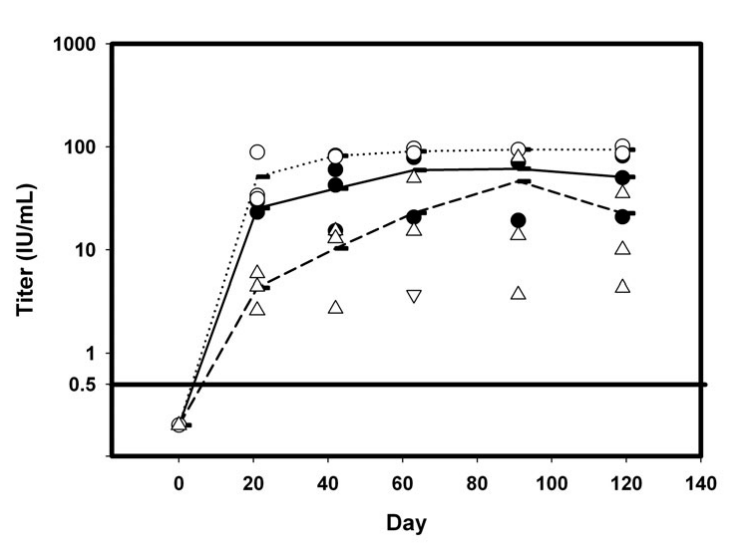
**Neutralizing Titers after Single Administration of PDNA:DMRIE-DOPE in rabbits**. Groups of three rabbits were vaccinated with either 300 μg (○; dotted line) or 10 μg (Δ; dashed line) of rabies G protein pDNA VR7203 as described in Materials and Methods. pDNA:cationic lipid molar ratio was kept at 4:1. Control group was vaccinated with IMRAB^® ^3(●; solid line). Lines connect mean titer values for each group at each time point. Protection threshold (0.5 IU/mL) is indicated with a horizontal line.

In order to evaluate the immunological response to a single pDNA administration, groups of three rabbits were vaccinated by a single intramuscular injection of either 300 μg or 10 μg of rabies G protein-expressing pDNA formulated with DMRIE-DOPE (4:1 DNA:cationic lipid ratio). A control group was injected with a commercial rabies veterinary vaccine, IMRAB^® ^3. Animals were bled at regular intervals and the rabies neutralization titers were obtained by RFFIT assay (Figure 2). All three groups had protective neutralizing titers (i.e., >0.5 IU /mL) 21 days after vaccination; high neutralizing titers were maintained for the duration of the study (120 days). Titers at day 21 were about an order of magnitude higher in the 300 μg pDNA dose group compared to the 10 μg dose group. Vaccination with 300 μg of DMRIE:DOPE-formulated pDNA resulted in neutralizing titers that were not statistically different from those obtained with the conventional rabies vaccine. Vaccination with the 10 μg pDNA dose resulted in protective titers about half a log lower than those obtained with the 300 μg pDNA dose at day 120 but still almost two orders of magnitude higher than the protective threshold (0.5 IU /mL) and not statistically different from titers obtained using the conventional vaccine.

### Vaxfectin™-formulated rabies G protein pDNA induces higher neutralizing titers than DMRIE-DOPE-formulated pDNA after single administration in mice

**Figure 3:**
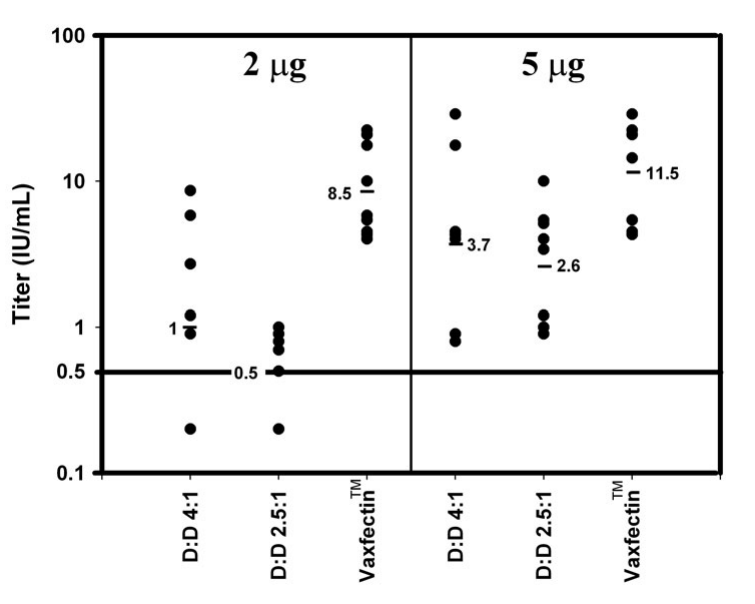
**Neutralizing Titers after single administration of cationic lipid-formulated pDNA in mice**. Data points indicate individual animal neutralizing titers 48 Days after vaccination with either 2 μg or 5 μg of cationic lipid-formulated pDNA. GMT values for each group are also indicated (▬). pDNA was formulated with either DMRIE-DOPE (D:D; 4:1 or 2.5:1 molar ratio) or Vaxfectin™ (4:1 molar ratio) as described in Materials and Methods. Protection threshold (0.5 IU/mL) is indicated with a horizontal line.

The differential effect of DMRIE-DOPE and Vaxfectin™ formulations on rabies neutralizing titers was explored by vaccinating mice with a single administration of cationic lipid-formulated pDNA. Two pDNA doses were used (2 μg and 5 μg); DMRIE-DOPE was formulated at a molar ratio (pDNA:cationic lipid) of either 2.5:1 or 4:1; Vaxfectin™ was formulated at a molar ratio of 4:1 (pDNA:cationic lipid). Neutralizing titers 48 days after vaccinating mice with Vaxfectin™-formulated VR7203 were statistically superior (p = 0.004) to those obtained from administration of pDNA:DMRIE-DOPE at either dose (Figure 3). In addition, at the 2 μg pDNA dose, titers obtained after administration of pDNA:DMRIE-DOPE at 4:1 ratio (DNA:cationic lipid) were marginally superior to the titers from administration at the 2.5:1 ratio; titers from the 4:1 and 2.5:1 ratios were statistically indistinguishable from each other at the 5 μg pDNA dose. Serum of all animals in the Vaxfectin™ group at the 2 μg dose showed protective neutralizing titers while up to 20% of the mice in the DMRIE-DOPE groups did not respond to the vaccination.

## Discussion

The value of cationic lipid formulations in enhancing humoral responses to pDNA vaccines is well documented [9,16,19]. Specifically, efficacy of a pDNA:DMRIE-DOPE rabies vaccine (2.5:1 pDNA:cationic lipid molar ratio) was convincingly demonstrated by Fischer and co-workers using a horse model. Development of a pDNA rabies vaccine for human use requires evaluation of improved formulations including alternative cationic lipid systems such as Vaxfectin™ and the determination of optimal pDNA:cationic lipid ratios. Our data provide some insight into possible formulation improvements using two different cationic lipid systems. We also provide data comparing the immunogenicity of rabies pDNA to a commercially available inactivated virus vaccine.

Neutralizing titers obtained after vaccination of mice with either 2 or 5 μg of cationic lipid-formulated pDNA indicate that Vaxfectin™ can improve the immunological response compared to DMRIE-DOPE and therefore represents a good candidate for formulation of a rabies pDNA vaccine, and potentially other vaccines that depend primarily on an antibody response for protection. Neutralizing antibody levels in the pDNA:Vaxfectin™ mice groups were statistically superior to those observed in the pDNA:DMRIE-DOPE groups at either 4:1 or 2.5:1 molar ratios. The difference between Vaxfectin™ and DMRIE-DOPE was emphasized at the lower dose (2 μg; p = 0.004) while still being statistically significant at the 5 μg dose (p = 0.009). Interestingly, the neutralizing antibody titers for the DMRIE-DOPE groups formulated at the 4:1 molar ratio were consistently higher than for the 2.5:1 molar ratio groups. Again, the difference between the DMRIE-DOPE groups was accentuated at the lower pDNA dose. The difference between the GMTs (Geometric Mean Titer) of the two DMRIE-DOPE ratios used is statistically marginal and will require further investigation. It is important to note that we present neutralizing antibody titers for one time point only (48 days after vaccination) since relative differences in GMT could change over time. However, our data indicate that formulation of pDNA with Vaxfectin™ elicits fast and robust humoral responses to rabies G protein. In addition, all mice in the Vaxfectin™ groups had antibody levels well above the protective threshold (0.5 IU/mL) while there were non-responder animals in the DMRIE-DOPE groups at the low dose (2 μg).

Rabbit immunogenicity data presented here confirm that high persistent neutralizing titers can be obtained with low pDNA doses. In fact, a single dose of 10 μg of DMRIE-DOPE-formulated pDNA elicited neutralizing titers almost two orders of magnitude over the protective threshold (0.5 IU/mL) that persisted for at least 120 days after administration. The magnitude and duration of the response were equivalent to that obtained with a single administration of a traditional rabies veterinary vaccine. The upper limit of the humoral response to DMRIE-DOPE-formulated pDNA in the rabbit was determined by administering three 1 mg doses. The antibody titers obtained after this vaccination regimen reached 100 IU/mL by day 41 and did not increase after the day 56 booster injection. High titers were maintained for the duration of the study (195 days). The magnitude of this response was statistically equivalent to that obtained after a single injection of 300 μg of the cationic lipid-formulated pDNA vaccine. The kinetics of the response after a single administration of 10 μg of pDNA were slower than those observed after administration of 300 μg of the vaccine. Titers at day 21 were about one order of magnitude lower for the 10 μg dose group compared to the 300 μg group. However, titers were statistically equivalent by day 91 and stayed comparable for the duration of the study. This latter observation suggests that the dose-dependent kinetics of the response need to be considered when designing a vaccine for post-exposure applications, as lower doses could be used for vaccines intended for pre-exposure prophylaxis.

In conclusion, our data suggest that the choice of cationic lipid and the optimization of pDNA:cationic lipid ratios can offer significant enhancement of the humoral response to rabies G protein and potentially other antigens. Specifically, the use of Vaxfectin™ in a rabies pDNA formulation may increase effectiveness while allowing for delivery of low doses of pDNA. The results of this research provide valuable information for development of a rabies pDNA vaccine for the developing world.

## Abbreviations

bp, base pairs; GMT, Geometric Mean Titer; IU, International Unit; PBS, phosphate buffered saline; pDNA, plasmid DNA
